# The Impact of Diet on Body Composition in a Cohort of Pediatric and Adult Patients with Maple Syrup Urine Disease

**DOI:** 10.3390/nu16183145

**Published:** 2024-09-18

**Authors:** Roberta Pretese, Cristina Bonfanti, Martha Caterina Faraguna, Marialetizia Fantasia, Viola Crescitelli, Silvia Barzaghi, Mara Botti, Giulia Mezzanotti, Serena Gasperini

**Affiliations:** 1 Department of Pediatrics, Fondazione IRCCS San Gerardo dei Tintori, 20900 Monza, Italy; roberta.pretese@irccs-sangerardo.it (R.P.); cristina.bonfanti@irccs-sangerardo.it (C.B.); m.faraguna@campus.unimib.it (M.C.F.); marialetizia.fantasia@irccs-sangerardo.it (M.F.); viola.crescitelli@irccs-sangerardo.it (V.C.); silvia.barzaghi@irccs-sangerardo.it (S.B.); 2 Residency in Pediatrics, University of Milano Bicocca, 20126 Milano, Italy; 3 Rare Disease Centre, Fondazione IRCCS San Gerardo dei Tintori, 20900 Monza, Italy; mara.botti@irccs-sangerardo.it; 4 Deparment of Biology Applied to Nutrition Sciences, University of Milano Statale, 20122 Milano, Italy; nutrizionistamezzanotti@gmail.com

**Keywords:** maple syrup urine disease, branched-chain amino acids, body composition, hypoproteic diet, osteopenia, bone mineral density

## Abstract

The treatment for Maple Syrup Urine Disease (MSUD) consists of a hypoproteic diet with integration therapy to limit leucine intake, ensuring adequate energy, macronutrients, and micronutrients to prevent catabolism and promote anabolism. We conducted a retrospective cross-sectional study at the Metabolic Rare Disease Unit, Fondazione IRCCS San Gerardo dei Tintori, Monza, Italy. Patients with MSUD who were over 3 years old, not treated with liver transplantation, and who provided written consent, were included. The study aimed to describe the dietary treatment of patients with MSUD, evaluate growth data, and analyze the effect of a low-protein and semi-synthetic diet on body composition. Data on height, weight, BMI, waist circumference, food intake, physical activity, and DEXA scans were collected. Thirteen subjects (11 classic MSUD, 2 intermediate MSUD) were included, of which 5 < 18 years old. Results indicated that patients with MSUD follow a balanced diet and have body compositions like healthy subjects in terms of fat and lean mass. A high incidence of osteopenia was observed from a young age, with a positive correlation between protein intake and lean mass and a negative correlation between BCAA-free mixture consumption and bone mineral density z-score. The study highlights the positive effects and potential consequences of the semi-synthetic diet on the body composition of patients with MSUD. A similar study involving all Italian metabolic centers treating MSUD is recommended.

## 1. Introduction

Maple Syrup Urine Disease (MSUD) is an autosomal recessive hereditary metabolic disorder caused by reduced activity of branched-chain α-ketoacid dehydrogenase (BCKD), a mitochondrial multienzyme complex that catalyzes the oxidative decarboxylation of branched-chain ketoacids in the second step of the catabolic pathway of the branched-chain amino acids (BCAA) leucine, isoleucine, and valine [1]. Its incidence is 1:185,000 live births [1], although recently published Italian newborn screening data reported a lower incidence in Italy (1:201,000) [2]. There are four metabolic phenotypes of MSUD defined by the residual enzyme activity of BCKD [3]. In order of decreasing severity, MSUD is classified into classic, intermediate, intermittent, and thiamine-responsive phenotypes [1,3].

The mainstay of treatment for MSUD is a hypoproteic diet, which limits the intake of leucine (LEU) [4], associated with a supplementation therapy, which provides energy, macronutrients, and micronutrients adequate based on sex and age in order to prevent catabolism and encourage anabolism [1]. The MSUD diet depends on the patient’s clinical condition; each patient is provided with a diet for a state of well-being, as well as an emergency diet [4]. Therefore, the diet is continuously modified based on the BCAA values detected in the plasma.

Given the negative impact on quality of life from following such a restrictive diet, combined with the risk of neurological deterioration due to acute leucine intoxication, liver transplantation is a valid therapeutic option for the classic form of MSUD [1].

The diet therapy involves integration with BCAA-free amino acid formulas since the amount of leucine tolerated is very low and the natural proteins taken do not cover the daily protein needs; the integration provides an intake of about 1–1.5 g/kg/day of amino acids [5]. These formulas are often enriched, even in micronutrients, to guarantee a balanced diet. Valine (VAL) and isoleucine (ILE) may be supplemented (between 50 and 300 mg/day) as elements necessary for protein synthesis. To avoid deficiencies, it is desirable to maintain plasma concentrations at values slightly higher than the norm [4]. Most of the carbohydrate, lipid, and caloric requirements are provided by naturally protein-free foods and commercial low-protein foods.

MSUD dietary therapy aims at ensuring metabolic stability and adequate growth, thus achieving an ideal body composition. There are few studies in the literature analyzing dietary therapy and body composition in patients with MSUD, with most analyzing inborn errors of intermediary metabolism [6,7]. De Castro et al. found that subjects with intermediary inborn errors of metabolism (heterogeneous diseases such as aminoacidopathies, carbohydrate disorders, and fatty acid beta-oxidation disorders) have higher body mass index (BMI) and waist circumference values and lower levels of bone mineralization compared to healthy controls, while no significant differences in terms of fat mass and muscle mass are described [6]. On the contrary, Campo et al. state that patients with MSUD do not show significant differences in fat mass and lean mass compared to controls [8].

Regarding bone mineralization, a recent study demonstrated a high incidence of orthopedic problems in patients affected by organic acidurias and MSUD [9]. The reason is probably multifactorial, including a low-protein and semi-synthetic diet, calcium and vitamin D deficiencies, reduced physical activity, and a recurrent state of metabolic acidosis that can determine reduced bone mineralization [7,10,11,12].

The aim of this study was to describe the dietary treatment of patients with MSUD, both adults and children, to compare its bromatological characteristics to that of healthy subjects, to evaluate auxological data for patients with MSUD, and finally, to analyze the possible effects of a low-protein and semi- synthetic diet on body composition.

## 2. Materials and Methods

### 2.1. Study Design

A retrospective/observational cross-sectional and descriptive study was carried out at the Metabolic Rare Disease Unit of the Fondazione IRCCS San Gerardo dei Tintori, Monza, Italy.

The inclusion criteria were a diagnosis of MSUD using biochemical and genetic data, follow-up at our Center, and provision of informed consent by the patient or their legal guardian. Exclusion criteria were age < 3 years, previous liver transplantation, denial of consent, and comorbidities that could affect bone health.

Data were collected between March 2021 and January 2022.

The study was approved by the Comitato Etico Territoriale Lombardia 3, protocol number 3984.

### 2.2. Auxological Parameters

During regular follow-up, weight, height, body mass index (BMI), and waist circumference were measured for each patient.

The weight was measured using an analytical balance (SECA) with a precision of 0.01 kg and the recordings were taken with an accuracy of 0.1 kg. A wall-mounted stadium meter (SECA) was used to measure the height with an accuracy of 0.1 cm. Then, the body mass index (BMI) was calculated (BMI = weight in kg/height^2^ in meters). Center for Disease Control and Prevention (CDC) percentiles were used to classify the pediatric patients’ data: subjects with a BMI < the 85th percentile are considered of normal weight, subjects with a BMI between the 85th and 95th percentiles are overweight, and those with a BMI > the 95th percentile are obese. In adults, a BMI ≥ 25 kg/m^2^ and a BMI ≥ 30 kg/m^2^ are defined as overweight and obese, respectively.

Measurement of waist circumference (WC) was carried out in an upright position, at the end of an exhalation, by placing the flexible centimeter at the midpoint between the lower costal margin and the anterior–superior iliac crest, with an accuracy of 0.1 cm. In European adult women, a WC > 80 cm is an index of central obesity, while in men it is a WC > 94 cm. For the determination of central adiposity in pediatric subjects, the ratio between waist circumference and height was used (Waist Circumference/Height Ratio, WCHr). WCHr values > 0.5 indicate an increased cardiovascular risk. The trunk-to-limb ratio was used as a marker of central obesity in adult patients; the trunk-to-limb ratio (TLr) is obtained from the ratio between trunk fat mass and limb fat Mass.

### 2.3. Biochemical Parameters

During scheduled clinical check-ups (biannual for children, annual for adults), liver and kidney function parameters (aspartate transaminase, alanine transaminase, creatinine, urea), bone metabolism parameters (calcium, vitamin D, phosphate, parathyroid hormone), and plasma amino acids were measured via a venous blood test. These values were recorded during the period of DEXA execution. For the BCAAs, the annual average was calculated and the average DEXA for the year was considered here.

### 2.4. Body Composition Analysis

Body composition was analyzed by the Total Body Dual Energy X-ray Absorptiometry (DEXA) using the HOLOGIC^®^ DENSITOMETERS Auto Whole-Body Fan Beam instrument, discovery model, at the Italian Auxological Institute IRCCS in Milan. Among the parameters detectable by DEXA, the following were evaluated in this study: the total lean mass (LBM, in grams), from which the lean body mass index (LBMI) was obtained by calculating the ratio between the total lean mass (kg) and the square of the height (m^2^); total adipose mass (FM, in grams), from which the fat mass index (FMI) was obtained by calculating the ratio between the total adipose mass (kg) and the square of height (m^2^); total body fat mass (expressed as a percentage of body weight, FM%); trunk fat mass (in grams) indicated as a percentage of total trunk body mass (Trunk FM%) for pediatric patients and as a trunk-to-limb ratio fat mass for adults, and total bone mineral density (BMD, in grams).

These indices were compared with reference intervals for black and white populations, derived from The National Health and Nutrition Examination Survey (NHANES) and FMI percentiles for the pediatric population [13,14]. For pediatric subjects, trunk fat mass values > +1 SD and <−1 SD were considered pathological [15], while for all subjects, values > +2 SD or <−2 SD from the mean were considered pathological.

BMD measurements were expressed as BMD z-scores. Osteoporosis was defined as a z-score > −2.5, and osteopenia as a z-score between −1 and −2.5 [16,17,18].

### 2.5. Nutritional Analysis

A two-day food diary was used to evaluate the total energy intake and the intake of macro- and micro-nutrients for each subject, with particular attention paid to leucine intake. The food diary was completed by the caregiver for children, while for adults it was completed by the patient themselves or by the caregiver in case of intellectual disability.

The bromatological composition data of the diet were expressed as mean ± standard deviation (SD). In case of an inability to compile a food diary, a 24 h recall was used instead [19,20].

The data collected were used to evaluate nutritional adequacy through comparison with the reference levels for the Italian population (LARN) [21].

### 2.6. Physical Activity

The level of physical activity of the patients was calculated using the International Physical Activity Questionnaire (IPAQ) [22]. The pediatric patients’ scores ranged from 1 to 5 and were classified as 1–1.7 (low physical activity), 1.7–3.4 (moderate physical activity), and 3.4–5 (intense physical activity). The adult scores were expressed in MET (Metabolic Equivalent Task, metabolic equivalent of activity). A score < 600 METs indicated low physical activity; 600–1500 METs indicated moderate physical activity, and >1500 METs indicated intense physical activity.

### 2.7. Statistics

Correlations were calculated using Pearson’s correlation coefficient; then, a simple linear regression was performed to study the correlation between protein intake and lean body mass index and between consumption of BCAA-free mixtures and bone mineral density.

Statistical significance was set at *p* < 0.05.

The software STATA 16 (StataCorp—College Station, TX, USA) was used for statistical analysis.

## 3. Results

### 3.1. Clinical Characteristics of the Population

A total of 13 patients with MSUD were enrolled in the study; 11/13 were affected by the classic form and 2/13 by the intermediate form. In the classic form group, 36% were children (<18 years old; 4/11, mean age 12.3 years), and 64% were adults (7/11, mean age 27.3 years). The intermediate form group consisted of one child and one adult, 17 and 37 years old, respectively.

All subjects were diagnosed after metabolic decompensation before newborn screening (NBS) for MSUD was introduced in Italy.

Of the whole sample, 15% (2/13) had serious disabilities and could not walk independently. These patients suffered from neonatal metabolic decompensation, which led to white matter damage and dystonic diplegia.

### 3.2. Diet

No patient in the cohort was usually fed by a nasogastric tube.

In 11/13 (85%) patients, a food diary for two non-consecutive days was filled in, while in 2/13 cases (15%) a 24 h recall was performed due to an inability to fill in the food diary.

All patients with the classic form, both adults and children, used protein-free products, BCAA-free amino acid formulas, and valine and isoleucine supplementation. All patients counted the daily protein quota as leucine equivalents and used exchange lists with 50 or 100 mg of leucine. Natural proteins were provided from vegetables, small quantities of fresh cheeses, and dairy products. All patients had an emergency letter.

Patients with the intermediate form tallied their daily protein amounts using exchange lists with 2 g protein servings and used the BCAA-free amino acid formulas; they did not use valine and isoleucine supplements.

Comparing the bromatological composition of the patients’ diets to the reference levels for the healthy population (LARN 2014), it emerged that the diet was balanced in terms of macronutrients, even if carbohydrates and lipids were at the upper limits of the reference ranges.

In terms of protein, the daily intake was guaranteed, although this quota was mainly due to the intake of BCAA-free amino acid formulas. Such formulas fulfilled on average 80.6 ± 8% of the total protein requirement (1 g/kg/day) in children with classic MSUD and 85.6 ± 3.7% in adults; while for the intermediate form, the formulas fulfilled 29% in children (0.7 g/kg/day) and 65% in adults (0.5 g/kg/day).

The daily average intake of leucine in the classic MSUD group was 414 ± 117 mg/day in children and 384 ± 98 mg/day in adults.

The intake of simple sugars by children and adult subjects with MSUD was higher in comparison to healthy people and represented 10–15% of daily kilocalories.

The caloric consumption of the pediatric cohort was reduced per kilocalories per day, but it was adequate for adult patients.

The intake of polyunsaturated fatty acids was not sufficient in both adult and pediatric patients.

### 3.3. Anthropometric Measurements and Body Composition

Weight, height, and BMI were obtained for all patients except one adult patient with the classic form. Of the pediatric patients with the classic form, 75% (3/4) were of normal weight and 25% (1/4) were overweight; 67% (4/6) of adults with classic MSUD were of normal weight, 16% were overweight (1/6), and 16% were obese (1/6). Both pediatric and adult patients with the intermediate form were of normal weight.

WC was measured in all patients except three adult patients with classic MSUD. Of the pediatric patients with the classic form, 50% (2/4) had a WCHr > 0.5; 25% (1/4) of adults with classic MSUD had a WC higher than the cutoff. Pediatric and adult patients with the intermediate form had normal WCHr and WC values.

All the pediatric subjects, the adult with intermediate MSUD, and five adults with classic MSUD accepted to undergo DEXA. The data obtained are reported in Table 1 and Table 2.

All subjects presented normal fat mass (FM), even though two pediatric patients were situated at the upper limit (z-scores of 1.79 and 1.82). The fat mass index (FMI) was within normal limits for all adult subjects, while 50% (2/4) of children with classic MSUD had an FMI ≥ 75th percentile.

All the patients evaluated presented reduced values of lean body mass index (LBMI); one pediatric patient, especially, had a z-score of −2.3.

Fifty percent (2/4) of the pediatric patients with classic MSUD had pathological percentages of trunk fat, while all adults with both forms of the disease were within the normal range for the trunk-to-limb ratio.

Regarding bone mineral density (BMD), 25% of pediatric patients with classic MSUD presented normal values, while 75% had osteopenia. The pediatric patient with the intermediate form had normal values.

One patient (#2) suffered a bone fracture (long bone of the arm).

All adult patients (100%) with both classic and intermediate MSUD presented with osteopenia, and none had osteoporosis.

Two patients (#10 and #13) suffered bone fractures (rotula, long bone of the leg), of which one patient did not undergo a DEXA scan.

A worsening of the BMD z-scores can be observed between adult and pediatric patients with classic MSUD.

**Figure 1:**
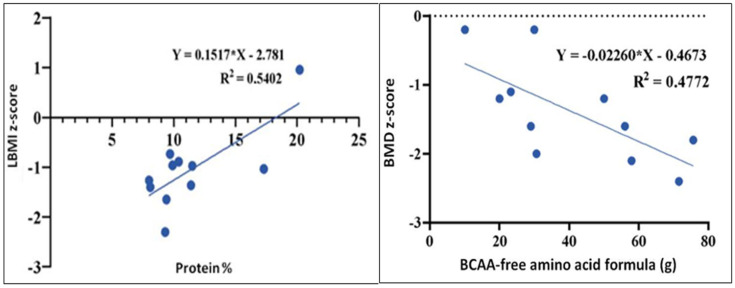
(**Left**). Correlation between protein consumption (expressed as percentage of total kcal) and LBMI z-score. (**Right**). Correlation between the intake of BCAA-free amino acid formulas (g/day) and BMD z-score. Each blue dot represents a patient.

The correlation between protein consumption (expressed as a percentage of total kcal) and LBMI z-score was studied; a significant difference (r = 0.7350, *p* = 0.01) between the percentage of protein intake and LBMI z-scores in subjects affected by MSUD was found. Using linear regression analysis, it can be observed (Figure 1, left) that when protein intake increased by one percentage point, the LBMI z-score increased by 0.15 points; the increase was significant (*p* = 0.01). The model obtained can be considered satisfactory on average (R^2^ = 0.5402).

Finally, the correlation between the amount of BCAA-free amino acid mixture taken daily (expressed in g) and the BMD z-score was evaluated. A negative correlation was observed for these variables (r = −0.69, *p* = 0.02). Linear regression analysis revealed that when BCAA-free blend intake increased by one unit, the BMD z-score decreased by −0.023; this increase was significant (*p* = 0.02). The model proposed is a discrete fit model (R^2^ = 0.48) (Figure 1, right).

### 3.4. Calcium-Phosphate Metabolism and Plasmatic Amino Acids

Table 3 reports the biochemical data for the calcium–phosphate metabolism at the time of the DEXA scan. All pediatric and adult patients presented values within the range; except for 2/8 of adults who had slightly below normal vitamin D levels with normal parathyroid hormone (PTH) levels. All patients took vitamin D supplementation at the time of the blood tests.

The children exhibited a good profile of BCAA; 1/5 showed an average leucine level above the guideline reference range [3]. Fifty percent (4/8) of adults presented elevated leucine levels; patients #2 and #3 presented good cognitive outcomes. For reference values, we adopted those from the guidelines for MSUD by Frazier et al., 2014 [4], which consider for a patient > 5 years old a leucine target of 75–300 µmol/L and valine and isoleucine targets of 200–400 µmol/L.

### 3.5. Physical Activity

The International Physical Activity Questionnaire was filled in by all patients (or their legal guardians) except one adult with the classic form. The results showed that pediatric subjects with both forms of MSUD had on average moderate physical activity (2.55 ± 1.1 and 1.8, respectively). Adult subjects with the classic form had on average low physical activity (MET 255 ± 414), while the patient with an intermediate form had a high degree of activity (>16,000 MET).

## 4. Discussion

Patients affected by MSUD are currently treated with a highly hypoproteic and semi-synthetic diet with supplementation of isoleucine and valine. Dietary treatment aims at ensuring metabolic stability and promoting normal growth and good living conditions [7]. For this reason, the daily quota of natural proteins allowed is only a few grams.

There are reference values for the dietary treatment of MSUD; in addition, the diet must be personalized and adapted to each patient. Each center relies on its previous clinical experience and on periodical monitoring of plasmatic amino acids, in particular leucine, based on which the diet is tailored for each patient.

Because of this evidence, and given the lack of information in the literature, we considered it appropriate to carry out an analysis of the diet and body composition of patients with MSUD to verify whether they could potentially be at risk of complications due to an imbalanced diet and the consumption of semi-synthetic products.

The studies published so far regarding body composition in diet-restricted subjects with inborn errors of intermediate metabolism (including MSUD) often have conflicting results [8,22]. In particular, de Castro et al. reported that patients with amino acid metabolism disorders had a higher level of fat mass and lower BMD z-scores than healthy controls [6], while a study conducted exclusively on patients with MSUD demonstrated that there are no differences between the body composition of subjects with MSUD and healthy subjects [8].

In this study, we found that on average, both adult subjects and pediatric patients showed a normal body composition, with values within the physiological ranges for both FM% and LBMI. LBMI z-scores correlated positively with total protein intake; this probably demonstrates that, despite the minimal amount of natural proteins taken, the BCAA-free amino acid mixtures contribute to the stability of lean mass.

Published data describe that subjects affected by rare metabolic diseases who follow a hypoproteic diet have a greater accumulation of fat in the truncal area [10]. In this study, however, we did not find a high percentage of truncal fat.

Regarding bone health, 70% of this cohort was affected by osteopenia. A worsening trend was observed between the pediatric and adult patients with classic MSUD. The explanation of such findings is multifactorial.

First, BCAA-free mixtures could have a negative effect on bone health. In fact, in this study, the results obtained from the DEXA analysis showed a high percentage of osteopenia in both adults and children (although of a young age), as well as a negative correlation between the intake of the BCAA mixture and BMD z-scores. Such findings were highlighted as well by Walter and MacDonald, who found problems such as osteoporosis and failure to thrive in some patients; however, the causes are not yet known [23].

In our clinical practice, to try and counter such findings, all patients are supplemented with vitamin D and are encouraged to exercise regularly [6].

Second, previously published data support the relationship between the excessive intake of simple sugars, especially those from common sugary drinks, and reduced bone mineralization [24,25]. It is interesting to note that in the population studied, many subjects had high consumption of these drinks as well as other foods rich in simple sugars.

Third, the homeostatic role of the bone as a buffer for acid is known [26]. It has been demonstrated that during states of metabolic acidosis, the bone appears to be decisive in maintaining a physiological systemic pH at the expense of bone mineral content being depleted. Rovelli et al. found metabolic acidosis in 60% of patients with phenylketonuria (PKU), and venous blood gas pH was significantly correlated with BMD z-scores [12].

Fourth, it is known that physical exercise plays an important role in body composition, influencing FM and BMD. De Castro and colleagues have, in fact, demonstrated that physical activity correlates positively with bone mineralization levels [6]. In our cohort, questionnaires on physical activity suggested that, on average, the subjects performed medium–low daily physical activities.

Finally, in this cohort, we did not find an increase in BMI, even though 30% of patients were overweight/obese. A high consumption of simple sugars by children and adults with MSUD was found, representing 10–15% of daily kilocalories. Such intake of simple sugars is placed at the upper limits with respect to the recommendation provided by the LARN 2014, while for the World Health Organization (WHO), which limits the consumption of simple sugars to less than 10% of daily calories, both pediatric and adult subjects exceeded expected levels.

In our population, many subjects had high consumption of sugary drinks, which is related to poorer bone health [24]. In fact, children in particular mixed the BCAA-free blend with sweet/sugary drinks to improve palatability and diet adherence. In addition, the glucolipid mixtures, which are prescribed to guarantee the daily caloric needs without increasing proteins, contribute to the increase in daily sugars.

Further, when patients are ill, the intake of carbohydrates, sugars, and fats is implemented in order to increase the caloric intake to avoid catabolism. In the long term, such behavior can contribute to excess weight, leading to obesity.

In these patients, it would be advisable to perform calorimetry to measure how many calories are needed precisely to obtain a good compensation and, at the same time, to prevent the patient from becoming overweight and developing various problems related to obesity in adulthood.

The strength of this study is that the cohort consists only of patients with MSUD who were all treated at the same center, with a comparable dietary therapy tailored to the specific form of the disease. This is challenging for hereditary metabolic diseases due to their low incidence. At the same time, we would like to extend these first results and perform a multi-center study. Furthermore, dietary, lifestyle, biochemical, and radiological data were analyzed, providing a thorough analysis.

The limitations of this study are that calorimetry was not performed, nor was the correlation with acidosis and low bone mineralization studied. Furthermore, the population was heterogeneous in age and severity of the disease. On the contrary, the highlight of this study is that by analyzing both pediatric and adult patients, one can see the worsening of bone health in adulthood after a lifetime of the same diet therapy.

## 5. Conclusions

The data obtained made it possible to analyze the food intake of subjects with MSUD, highlighting a good adherence to the indications provided by the dietician, especially in the children’s group. At the same time, it was possible to highlight the lack of polyunsaturated fatty acids, for which integration has been hypothesized.

The DEXA analysis showed that, on average, the semi-synthetic diet consumed by patients with MSUD does not compromise their body composition in terms of fat mass and lean mass. Regarding bone mineral density, however, results showed a strong tendency of these subjects to develop osteopenia, starting from a young age; the adult patients with classic MSUD had worse BMD in comparison to the children. This evidence requires future studies to investigate the potential risk factors, in particular whether the chronic intake of amino acid mixtures compromises the physiological mineralization process and whether it is appropriate to intervene with the administration of drugs that do not modify the pH of the protein substitutes, as well as hypothesizing the formulation of new mixtures that have a lower impact on bone health—the GMP-based mixture for phenylketonuria is an example.

It would be desirable to conduct a similar study on a national scale, involving all the Italian metabolic centers that treat patients with MSUD.

In conclusion, we believe this work should be continued by the SIMMESN (Italian Society for the Study of Hereditary Metabolic Diseases and Neonatal Screening) dietetic and nutrition working group.

## Figures and Tables

**Table 1:** Parameters and indices obtained from the DEXA analysis of each individual pediatric patient with classic and intermediate forms (with relative standard deviations with respect to the reference values based on sex and age). Abbreviations: FM%, fat mass percentage; FMI, fat mass index; Pc, percentile; LBMI, lean body mass index; TLr, trunk-to-limb fat mass ratio; BMD, bone mineral density. Asterisk indicates patients who suffered from a bone fracture.

Pediatric Classic	Age	FM%	FM% z-Score	FMI	Pc FMI	LBMI	LBMI z-Score	Trunk FM%	Trunk FM% z-Score	BMD z-Score
1	13.5	32.1	0.11	4.95	10–25°	9.98	−2.30	22.8	−0.28	−1.6
2 *	14.0	34.4	0.45	6.77	25–50°	12.19	−1.36	30.5	0.65	−1.2
3	10.2	40.1	1.79	7.56	75–90°	10.85	−1.65	37.5	1.49	−0.2
4	8.2	36.7	1.82	7.01	75–90°	11.54	−1.27	35.9	1.59	−1.1
Pediatric intermediate	Age	FM%	FM% z-score	FMI	Pc FMI	LBMI	LBMI z-score	Trunk FM%	Trunk FM% z-score	BMD z-score
5	17.4	31.4	−0.16	6.06	25°	12.47	−1.40	22.8	−0.28	0.2

**Table 2:** Parameters and indices obtained from the DEXA analysis of each individual adult patient with classic and intermediate forms (with relative standard deviations with respect to the reference values based on gender and age). Abbreviations: FM%, fat mass percentage; FMI, fat mass index; LBMI, lean body mass index; TLr, trunk-to-limb fat mass ratio; BMD, bone mineral density. Asterisk indicates patients who suffered from a bone fracture.

Adult Classic	Age	FM%	FM% z-Score	FMI	FMI z-Score	LBMI	LBMI z-Score	TLr	TLr z-Score	BMD z-Score
6	27.8	38.4	0.33	9.03	0.03	13.79	−0.97	0.73	−0.34	−2
7	34.2	24.7	−0.16	6.05	−0.26	17.74	−0.73	1.16	0.49	−2.1
8	32.8	31.6	0.94	8.28	0.54	17.34	−0.89	0.78	−1.38	−2.4
9	19.5	30.9	−0.48	6.27	−0.58	13.40	−1.03	24.4	−0.08	−1.8
12	27.2	46.4	1.43	16.01	1.84	17.84	0.96	0.82	0.13	−1.6
Adult intermediate	Age	FM%	FM% z-score	FMI	FMI z-score	LBMI	LBMI z-score	TLr	TLr z-score	BMD z-score
13 *	37.3	19.6	−1.17	4.44	−0.97	17.39	−0.96	1.01	−0.52	−1.2

**Table 3:** Calcium–phosphate metabolism parameters at the time of the DEXA scan in pediatric and adult patients, and plasmatic amino acids (the average DEXA scan of the year is reported). Abbreviations: alanine transaminase (ALT), aspartate transaminase (AST), parathyroid hormone (PTH).

Pediatric Classic	AST U/L (<32)	ALT U/L (<33)	Calcium mg/dL (8.4–10.2)	Phosphate mg/dL (2.5–4.8)	Vitamin D ng/mL (>30)	Parathyroid Hormone pg/mL (14.9–56.9)	Creatinine mg/dL (0.51–0.95)	Urea mg/dL (7–20)	Leucine (Average) uMol/L (75–300)	Isoleucine (Average) uMol/L (200–400)	Valine (Average) uMol/L (200–400)
1	13	19	9.9	4.2	34.5	23.2	0.5	14	91.5	193.9	343.5
2	8	19	9.4	3.6	30.8	51.8	0.6	14	343.6	144.2	116.0
3	32	37	9.9	4.8	41	14.8	0.5	18	295.1	227.0	341.6
4	23	36	9.8	3.9	34.6	25.3	0.6	18	107.7	499.2	1916.1
Pediatric intermediate											
5	8	14	10.3	3.6	27.7	35.0	0.8	14	212	99.6	281.3
Adult classic											
6	12	11	9.5	3.2	31	40.7	0.8	27	215	265	304
7	21	35	NA	3.8	35	36	0.9	19	536	152	180
8	19	17	9.6	2.8	43	35.7	0.8	20	488	244	320
9	33	32	9.9	3.9	44.3	51.4	0.7	22	82.6	275.7	335.9
10	26	29	91	2.9	34.5	19.5	0.9	19	52.1	190	163.4
11	17	14	10	2.9	23	23.9	0.8	21	675.3	161.6	312.6
12	17	13	9.4	NA	37.7	33.2	0.7	18	195	123	112
Adult intermediate											
13	22	20	9.3	2.3	24.8	37.1	1.1	20	465	179	447

## Data Availability

All data generated or analyzed during this study are included in this article. Further enquiries can be directed to the corresponding author.
